# Overstuffing in resurfacing hemiarthroplasty is a potential risk for failure

**DOI:** 10.1186/s13018-019-1522-1

**Published:** 2019-12-30

**Authors:** Pieter C. Geervliet, Jore H. Willems, Inger N. Sierevelt, Cornelis P. J. Visser, Arthur van Noort

**Affiliations:** 1 NoordWest Clinics, Department of Orthopedic Surgery, Shoulder Unit, Wilhelminalaan 12, 1815 JD Alkmaar, the Netherlands; 2 Spaarne Hospital, Spaarnepoort 1, 2134 TM Hoofddorp, the Netherlands; 3 Alrijne Hospital, Simon Smitweg 1, 2353 GA Leiderdorp, the Netherlands

**Keywords:** Resurfacing humeral head implant, Overstuffing, Shoulder, Radiographs, Revision

## Abstract

**Purpose:**

Literature describes the concern of an overstuffed shoulder joint after a resurfacing humeral head implant (RHHI). The purpose of this study was to evaluate inter-observer variability of (1) the critical shoulder angle (CSA), (2) the length of the gleno-humeral offset (LGHO), and (3) the anatomic center of rotation (COR) in a patient population operated with a Global Conservative Anatomic Prosthesis (CAP) RHHI. The measurements were compared between the revision and non-revision groups to find predictive indicators for failure.

**Methods:**

Pre- and postoperative radiographs were retrieved from 48 patients who underwent RHHI from 2007 to 2009 using a Global CAP hemiarthroplasty for end-stage osteoarthritis. This cohort consisted of 36 females (12 men) with a mean age of 77 years (SD 7.5). Two musculoskeletal radiologist and two specialized shoulder orthopedic surgeons measured the CSA, LGHO, and COR of all patients.

**Results:**

The inter-observer reliability showed excellent reliability for the CSA, LGHO, and the COR, varying between 0.91 and 0.98. The mean COR of the non-revision group was 4.9 mm (SD 2.5) compared to mean COR of the revision group, 8 mm (SD 2.2) (*p* < 0.01). The COR is the predictor of failure (OR 1.90 (95%Cl 1.19–3.02)) with a cut of point of 5.8 mm. The mean CSA was 29.8° (SD 3.9) There was no significant difference between the revision and non-revision groups (*p* = 0.34). The mean LGHO was 2.6 mm (SD 3.3) post-surgery. The mean LGHO of the revision group was 3.9 (SD 1.7) (*p* = 0.04) post-surgery. Despite the difference in mean LGHO, this is not a predictor for failure.

**Conclusion:**

The CSA, LGHO, and COR can be used on radiographs and have a high inter-observer agreement. In contrast with the CSA and LGHO, we found a correlation between clinical failure and revision surgery in case of a deviation of the COR greater than 5 mm.

**Trial registration:**

Institutional review board, number: ACLU 2016.0054, Ethical Committee number: CBP M1330348. Registered 7 November 2006.

## Introduction

The hemi resurfacing humeral head implant (RHHI) provides good clinical results for patients with gleno-humeral osteoarthritis [1–7]. The purpose of a RHHI is to restore the patient’s individual anatomy and the lateral offset of the proximal humerus while preserving the bone stock of the humeral head [8–10].

Sizing of the proximal humerus is generally preoperative estimated on the radiograph and definitely measured during surgery. Because of a deformed proximal humerus, surgeons often have difficulty to accurate assess the correct size of the implant and restoring the anatomy compared with stemmed arthroplasty [8, 10]. In literature, high rate of revision of the RHHI is a concern [11–13]. Alolabi et al. [8] found a possible relation with overstuffing; however, in literature, there is no definite correlation reported between overstuffing and revision.

This study was performed as an extension to an ongoing follow-up study in patients treated with uncemented Global Conservative Anatomic Prosthesis (CAP) (DePuy Synthes, Warsaw, IN, USA) hemi resurfacing shoulder prosthesis from 2007 until 2009 [13–16]. At the 5–8 years follow-up, our results are in line with other studies of a concerning high rate of revision [13, 16].

The aim of this current radiographic study was to evaluate the ability to restore humeral head anatomy and to determine the inter-observer reliability of the critical shoulder angle (CSA), length of the gleno-humoral offset (LGHO), and deviation of the center of rotation (COR) in a hemi RHHI.

Furthermore, with these measurements to find prognostic tools to predict poor functional outcome and the necessary of revision, first, we used the pre-operative CSA which assesses the possible association of implant failure due to rotator cuff failure or progressive glenoid erosion. Second, we measured the LGHO before and after surgery. Finally, with best-fit circle technique, we measured the deviation COR of the prosthetic humeral head from native anatomy after resurfacing humeral head arthroplasty.

All measurements were performed on the shoulders of patients operated for primary, end-stage gleno-humeral osteoarthritis with a Global CAP resurfacing hemiarthroplasty. The group consisted of patients who underwent a revision arthroplasty and patients with good patient reported outcome measures.

## Materials and methods

### Patient selection

Between 2007 and 2009, 48 shoulders were operated using a Global CAP uncemented resurfacing shoulder hemiarthroplasty at two regional hospitals in the Netherlands (Alrijne Hospital and Spaarne Hospital). This cohort consisted of 12 males and 36 females. All patients were operated on by two senior orthopedic surgeons (AvN or CV) specialized in shoulder pathology. The included 48 shoulders with only primary gleno-humeral osteoarthritis had intact rotator cuff, sufficient bone stock (>60%) of the proximal humerus, and type A1, A2 or B1 glenoid (Walch Classification [17]) as assessed on radiographs and magnetic resonance imaging (MRI) scans. Patients with severe fatty infiltration (Goutallier [18] grade 4), paresis of rotator cuff muscles, wound healing problems, neuromuscular pathologies, or active infections were excluded for this study.

### Surgical protocol

The orthopedic surgeons did not use radiological planning prior to surgery. All operations were performed via deltopectoral approach. Osteophytes present were removed, and the cartilage of the head was reamed guided by the anatomical neck of the humerus. Appropriately sized prosthesis was placed in patient own (retro) version and inclination. The prosthesis is available in five sizes, and each size has two heights to match the anatomy of the proximal humerus. No glenoid implants were used. Due to a hydroxyapatite coating, no cement was used for fixation. Digital pre- and postoperative radiographs were retrieved from the 48 shoulders. The postoperative treatment protocol was immobilization with an arm sling on the first day. Hereafter, active and passive movement supervised by a physiotherapist was allowed. After 6 weeks, free and active movement, respecting the patient’s pain threshold, was encouraged and supervised by a physiotherapist.

### Radiographic measurements

Radiographic measurements were performed to assess the critical shoulder angle (CSA), length of gleno-humeral offset (LGHO), and the center of rotation (COR). For reliable assessments, four independent observers performed the measurements: two senior musculoskeletal radiologists (SB and BdW) and two orthopedic surgeons (PG and JW) specialized in shoulder pathology and shoulder arthroplasty performed the measurements. All measurements were taken electronically on radiographs displayed on a PACS workstation (Cerner Corp. Kansas City, Missouri, USA). Patient characteristics and patient-reported outcomes and revisions were unknown to the assessors. The X-ray technique of the two hospitals was standardized; the patients were positioned standing with their back against the image receptor and the non-affected side was turned 35–45° away from the image receptor. The affected arm was flexed 90° in the elbow and the underarm was internally rotated. The angle of the beam was tilted 15–20° in the cranial caudal direction and was centered toward the shoulder joint.

This “true” antero-posterior radiographs were used to perform the measurements. The assessors used the preoperative radiographs and the 6 weeks postoperative radiographs. If the 6 weeks radiographs were insufficient for assessment, the 1-year postoperative radiographs were used instead.

### Critical shoulder angle

**Fig. 1:**
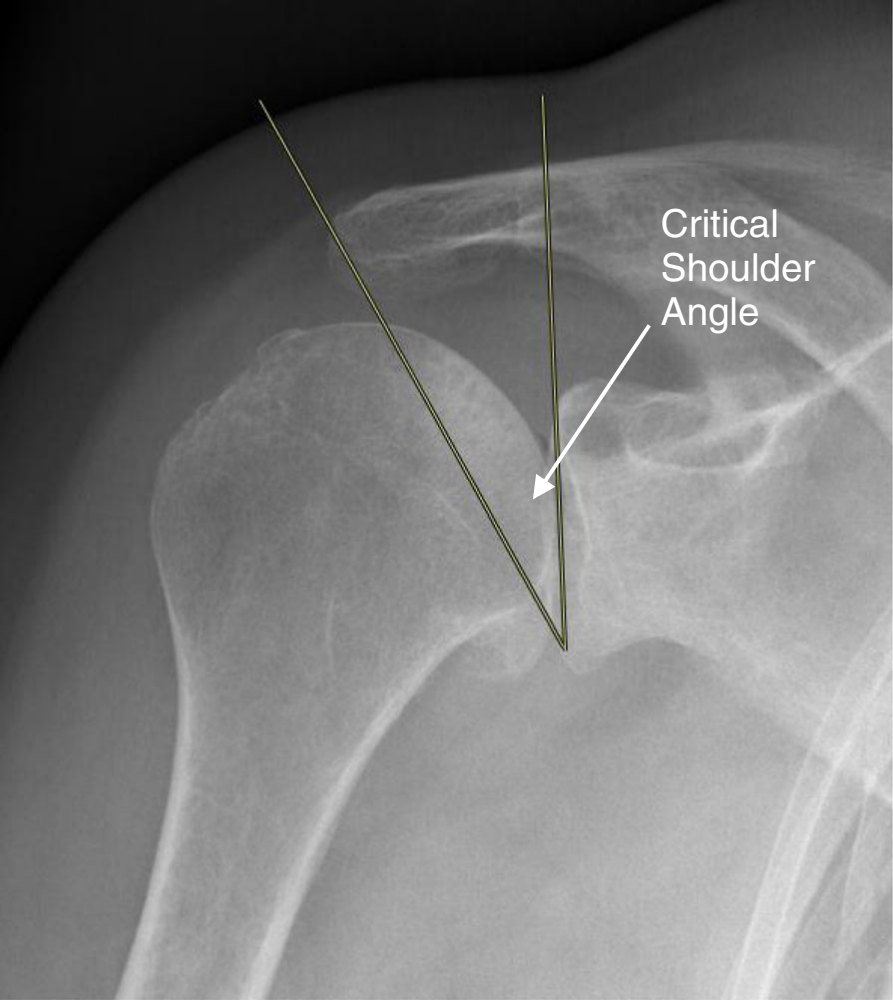
Radiograph of a right shoulder showing the assessment of the critical shoulder angle

The critical shoulder angle (CSA) was assessed on all preoperative “true” antero-posterior (AP) shoulder radiographs. The angle was formed by a line connecting the superior and inferior bony margins of the glenoid and a line drawn from the inferior bony margin of the glenoid to the most lateral border of the acromion (Fig. 1.) [19]. The CSA angle is defined by three grades (Table 1).

**Table 1:** Critical shoulder angle [19]

	Angle in degrees	
Grade I	< 30°	Osteoarthritis
Grade II	30°–35°	Normal
Grade III	> 35°	Rotator cuff tear

### Length of the gleno-humeral offset

**Fig. 2:**
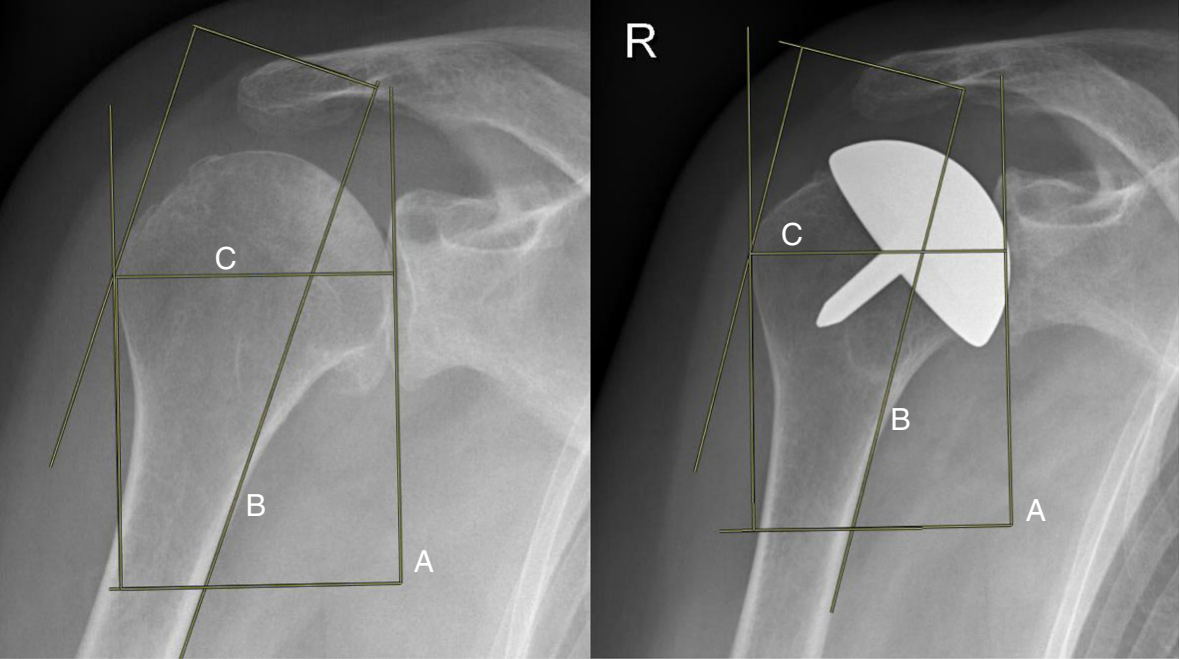
Radiograph of the right shoulder before and 6 months after implantation of the resurfacing hemiarthroplasty. The modified length of gleno-humeral offset is measured by drawing a line from the top to the bottom of the glenoid (**a**). The cortex of the humerus was drawn. A parallel line was shifted till it touched the most lateral cortex of the greater tubercle (**b**). The distance was marked as (**c**)

The modified length of the gleno-humeral offset (LGHO) of the 48 shoulders was assessed on both pre- and postoperative “true” AP radiographs [10, 20, 21]. First, a line from the top to the bottom of the glenoid cavity was drawn. Second, a parallel line was drawn from the center axis of the humeral bone until the most lateral part of the greater tubercle was touched. This point was marked and the perpendicular distance from the glenoid line to this point was noted as the modified measure of LGHO (Fig. 2). The length of the gleno-humeral offset is important in shoulder function, since it affects soft tissue tension and joint balancing. Normal LGHO averages from 54 to 57 mm (range 43–68 mm) [22]. As a result of gleno-humeral OA, with narrowing of the joint space, the soft tissue will adapt to the changed morphology. The LGHO should not increase after surgery [10].

### Center of rotation

The center of rotation (COR) was measured [8]. A best-fit circle was placed on the “true” AP radiograph using three preserved bone landmarks: the lateral cortex of the greater tubercle, medial calcar at the inflection point where calcar meets the articular surface, and the medial edge of the greater tubercle medial of the footprint of the supraspinatus tendon. A second circle, the implant matched circle, was placed to fit the curvature of the prosthetic humeral head. The COR was identified from each circle, and the distance between the CORs was calculated in millimeter (Fig. 3.1). A coordinate system was then generated from the anatomic COR, with the y-axis aligned parallel to the intramedullary axis and the x-axis defined as perpendicular to this line. This created four regions in which the location of the decimation of COR could be defined; superior medial, inferior medial, superior lateral, and inferior lateral (Fig. 3.2). By use of the COR, we measured the overstuffing of the shoulder joint after resurfacing shoulder prosthesis. Medial deviation of the COR was defined as overstuffing [8].

**Fig. 3:**
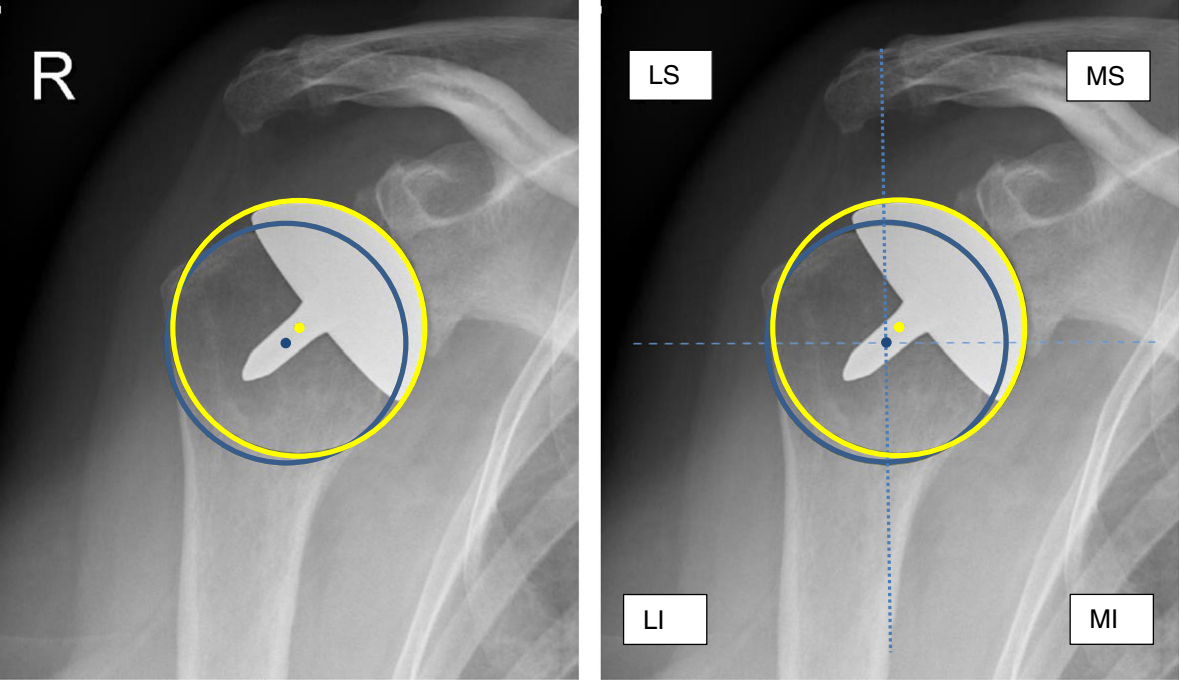
**1** Radiograph of a right shoulder. Anatomical circle with center of rotation (blue) and postoperative center of rotation (yellow). The distance in millimeters between the both centers was measured. **2** Radiograph of a right shoulder, demonstrating the anatomical circle (blue) and the implant matched circle (yellow) and the 4 quadrants. LS, lateral superior; MS, medial superior; MI, medial inferior; LI, lateral inferior

### Revision

At the 9-year follow-up (range 5–12 years), 12 shoulders (23%) had a revision to a total shoulder arthroplasty. One patient had a revision for pain and loss of range of motion. On the radiographs, there was progressive glenoid erosion. At revision to total shoulder arthroplasty (TSA), the tissue samples retained per-operatively were tested positive on *Pantoea agglomerans*, *Staphylococcus epidermidis*, and *Propionium acnes*. We excluded this patient from data analysis for infection reason. The 11 revision patients used in data analysis are mentioned in Table 2. All other revisions had negative peroperative (tissues obtained during surgery/revision operation).

**Table 2:** Revision of 11 patients

Reason	Revision	Comment
Glenoid erosion	TSA	Progressive pain
Arthrofibrosis	TSA	Pain and poor function
Severe glenoid erosion	TSA	Progressive pain
Rotator cuff arthropathy	RSA	Pain and poor function, traumatic rotator cuff tear, glenoid erosion
Rotator cuff arthropathy	RSA	Earlier surgical subscapularis tendon repair
Pain and poor function	TSA	Progressive pain and loss of range of motion, minimal glenoid erosion
Pain and poor function	TSA	Patient is emigrated, revision surgery was abroad
Severe glenoid erosion	RSA	Progressive pain
Glenoid erosion	TSA	Progressive pain
Glenoid erosion	TSA	Progressive pain
Severe glenoid erosion	RSA	Progressive pain and loss of range of motion

*TSA* total shoulder arthroplasty, *RSA* reverse shoulder arthroplasty

### Statistical analysis

Statistical analysis was performed by use of Statistical Package for the Social Sciences (SPSS) (IBM, Armonk, NY, USA, version 26.0). After confirmation of normal distribution, continuous variables are presented as means with standard deviations (SD). Categorical data are described as frequencies with accompanying proportions. Differences between the revision and non-revision group were assessed using Student’s *t* tests or chi-squared tests, where appropriate.

Inter-observer reliability was assessed by calculating of the intra-class coefficient (ICC agreement, two-way random effect model) [23]. An ICC >0.7 was considered as sufficient [24, 25]. Additionally, the standard error of measurement (SEM) was calculated as the square root of the within-subject variance (i.e., sum of the between measures variance and the residual variance) with accompanying smallest detectable difference (SDD) as 1.96*√2*SEM [26].

To identify predictors for revision, univariate logistic regression was performed for potential predictors, such as age, gender, CSA, LGHO, and COR. In the case of significant association (adjusted significance level of 0.10), the factors were entered in a multivariate logistic regression analysis. For all analyses, odds ratios (OR) with 95% confidence interval (95%CI) were calculated and presented.

To calculate an optimal cut-off value of the measurement that was significantly associated with revision in the final model, a receiver operating characteristic (ROC) curve analysis was performed. A bootstrapping procedure, drawing 1000 bootstrap samples, was used to estimate a standard error to provide a 95%CI around the cut-off value. As a measure of accuracy, the area under the curve (AUC) was calculated.

## Results

### Population

The average age of the patient population was 77 years (SD 7.5), and 36 patients out 47 were female (77%). The demographics and measurements of the revision and non-revision group for the CSA, LGHO, and COR are outlined in Table 3.

**Table 3:** Demographics and measurements

	Total (*n* = 47)	Revision (n = 11)	Non-revision (*n* = 36)	*p* value
Age, years, mean (SD)	76.6 (7.5)	74.8 (6.4)	77.1 (7.9)	0.39
Gender, *n* (%)				
Male	11 (23)	4 (36)	7 (19)	0.25
Female	36 (77)	7 (64)	29 (81)	
CSA, mean (SD)	29.8 (3.9)	30.8 (3.0)	29.5 (4.2)	0.34
CSA, *n* (%)				
< 30	26 (55)	5 (46)	21 (58)	0.66
30–35	16 (34)	5 (46)	11 (31)	
> 30	5 (11)	1 (8)	4 (11)	
LGHO pre-operative, mean (SD)	49.6 (5.0)	51.1 (4.0)	49.1 (5.3)	0.26
LGHO post-operative, mean (SD)	52.1 (4.9)	54.9 (4.4)	51.3 (4.8)	0.03
LGHO CFB (SD)	2.6 (3.3)	3.9 (1.7)	2.2 (3.6)	0.04
COR, mean (SD)	5.6 (2.7)	8.0 (2.2)	4.9 (2.5)	<0.01

*CSA* critical shoulder angle (degrees), *LGHO* length of the gleno-humeral offset (mm), *COR* center of rotation (mm), *CFB* change from baseline

### Reliability and measurement error

The inter-observer reliability showed excellent reliability for the CSA, LGHO pre- and postoperative, and the COR, varying between 0.91 and 0.98 (Table 4).

**Table 4:** Inter-observer reliability

	CSA	LGHO pre	LGHO post	COR
ICC (95%CI)	0.97 (0.95–0.98)	0.96 (0.93–0.97)	0.91 (0.85–0.95)	0.98 (0.96–0.99)
SEM	0.69	1.13	1.52	0.43
SDD	1.91	3.12	4.22	1.2

*CSA* critical shoulder angle (degrees), *LGHO* length of the gleno-humeral offset (mm)—pre- and postoperative, *COR* center of rotation (mm), *ICC* inter-observer reliability, *SEM* standard error of measurement, *SDD* smallest detectable difference

### Critical shoulder angle

Based on the study by Moor et al. [19], CSA values were classified into three grades: grade I CSA <30°, grade II CSA 30–35°, and grade III CSA >35° (Table 1). The mean CSA of 47 shoulders is 29.8° (SD 3.9). We found no significant difference in CSA between the revision group and non-revision group (*p* = 0.34) (Table 3).

### Length of gleno-humeral offset

The mean LGHO increased from 49.6 mm (range 37.6–60.4) before surgery to 52.1 mm (range 37.2–61.7) after surgery. The increase of the LGHO was significantly higher in the revision group compared to that in the non-revision group (*p* = 0.04). The preoperative LGHO was not significantly different between the two groups (*p* = 0.26). However, the postoperative LGHO of the revision group was significantly different compared to the non-revision group (*p* = 0.03), see Table 3.

### Center of rotation

The mean deviation of the postoperative resurfacing head COR from the anatomic COR for all 47 cases was 5.6 mm (2.7 SD).

The mean COR in the non-revision and the revision group was 4.9 mm (2.5SD) and 8.0 mm (SD2.2), respectively. This difference was significant (*p* < 0.01). Of the 47 shoulders, five implants (12%) had the COR shifted to medial inferior. The remaining 43 shoulders had the COR shifted to medial superior. All shoulders in the revision group (*n* = 11) had the COR shift to medial superior, meaning overstuffing of the joint.

### Predictors of revision

Univariate analysis revealed that post-operative LGHO and the COR were both significantly associated with revision. However, in the final model only, the COR remained as a predictor for revision with an OR of 1.90 (95%Cl 1.19–3.02), see Table 5.

**Table 5:** Predictors of revision

Univariate	OR (95%CI)	*p* value
Age	0.96 (0.87–1.05)	0.38
Gender	2.37 (0.54–10.40)	0.25
CSA	1.09 (0.92–1.30)	0.33
LGHO preoperative	1.09 (0.94–1.25)	0.26
LGHO postoperative	1.19 (1.01–1.41)	0.04
LGHO change from baseline	1.19 (0.94–1.49)	0.15
COR	1.90 (1.19–3.02)	0.01
Multivariate	OR (95%CI)	*p* value
LGHO postoperative	1.16 (0.95–1.43)	0.15
COR	1.91 (1.14–3.20)	0.02
Final model	OR (95%CI)	*p*-value
COR	1.90 (1.19–3.02)	0.01

*CSA* critical shoulder angle, *LGHO* length of the gleno-humeral offset, *COR* center of rotation, *OR* odds ratio

ROC analysis of the COR revealed a cut-off point for revision of 5.8 mm (95%Cl 4.0–8.4) with a corresponding AUC of 0.82 (95%CI 0.68–0.95).

## Discussion

Inaccurate sizing or positioning of a prosthetic humeral head can lead to overstuffing the joint and poor outcomes, including glenoid erosion, rotator cuff tearing, and, in the case of a glenoid component, wear and loosening [27–32].

We assessed the CSA, LGHO, and COR in a selected cohort of patients operated on with a Global CAP, an uncemented resurfacing shoulder hemiarthroplasty for primary end-stage osteoarthritis.

The aim of this study was to measure inter-observer reliability of the CSA, LGHO, and COR and to define parameters to predict failure. The purpose of the Global CAP, like many other RHHI, is to recreate the normal anatomical gleno-humeral relationship of the shoulder. As considered by Mechlenburg et al. [10] and Alolabi et al. [8], the RHHI might potentially overstuff the gleno-humeral joint.

We found a high inter-observer reliability for the CSA, this is in line with other studies on CSA measurements [33]. Moor et al. [19] classified a CSA angle <30° as gleno-humeral OA and a CSA >35° as rotator cuff tear. In our series, with the observed minimal detectable difference of 1.9°, this classification should be interpreted with caution. Viehöfer et al. [34] showed that a higher CSA requires more rotator cuff activity to preserve joint stability. This leads to higher risk of rotator cuff failure [35–37]. Additionally, Watling et al. [38] found a high CSA being associated with glenoid component loosening and failure. In our series, however, we did not find a significant association between CSA angles and revision.

Originally, the measurements of the LGHO is performed using the distance from the base of the coracoid process to greater tubercle [31, 39]. But this measure shows systematic errors in inter-tester reliability because it is difficult to locate the base of the coracoid process [31]. Due to the reported problems with inter-tester reliability of the standard LGHO measurements, we used the modified LGHO [10, 20, 21]. Because, it is possible that factors like direct postoperative intra-articular fluid or releases related capsular laxity might falsely increase the LGHO measurements, we used the 6 weeks or 1-year post-operative radiographs.

In theory, LGHO after surgery should be identical to LGHO before the shoulder morphology changed caused by arthritis without structural changes of the soft tissue. But as osteoarthritis progresses with narrowing of the joint space, destruction of the joint cartilage, and capsule tightening, the soft tissue adapts to the changed morphology by losing elasticity and the LGHO should not be increased after surgery [10, 20, 21]. This in contrast with current study where the mean change of baseline of the LGHO increased by 2.6 mm and 3.9 mm in the non-revision group and revision group, respectively.

Like Mechlenburg et al. [10] in our study the LGHO is not reproduced. Additionally, the difference between the postoperative LGHO between the revision and non-revision is significant (*p* = 0.03). Nonetheless, we found that the postoperative LGHO is not a predictor of revision. Conform the study by Stilling et al. [21], we found high inter-observer agreement.

Alolabi et al. [8] found in their study that 65.1% of the RHHI demonstrated an inadequate reaming of the humeral head, resulting in overstuffing of the gleno-humeral joint. In our study, we found 88% overstuffing in all shoulders and 100% overstuffing in the revision group.

Multiple studies use different cut-off points to define overstuffing of the gleno-humeral joint [8, 27–30, 32].

In these studies, they assessed no relation to an increase of COR to patient-reported outcomes or revision. Pearl et al. [40, 41] already showed in their computer simulation studies that the COR in RHHI have great difficulty matching the geometric dimensions of the native gleno-humeral anatomy. However, these measurements were done on cadaveric humerus, without relation to patient-reported outcomes or revision. And computer studies may not be directly comparable to the results of radiographic studies. Our results regarding RHHI are in line with Alolabi et al. [8], the normal gleno-humeral anatomy, regarding the COR, is not reproduced. We found a significant increase in COR in the revision group compared to the non-revision group. In other words, the probability of revision increases significantly with an increased COR. Overstuffing has always been a suspect for failure [8]. However, this has not been demonstrated in the literature before. In this current study, we have shown a relation between failure and overstuffing.

The main limitation of this study is the small study group. Because of the fact that this concerns to an ongoing study of the Global CAP, it provides valuable information of this uncemented RHHI. The rate of revision (23%) at 9 years follow-up in our cohort is high. We excluded the patient with low-grade infection for data analysis because the authors believe it is difficult to distinguish between pain caused by glenoid erosion or pain caused by low-grade infection.

Three questions arise why the rate of revision was higher compared by studies by Levy et al. [1, 2, 42, 43]. First, a number of revisions can happen when inexperienced surgeons perform few procedures. However, the surgeons in this cohort are specialized shoulder surgeons, in high volume shoulder hospitals, with experience in shoulder replacement/revision, shoulder arthroscopic procedures, and fracture osteosynthesis.

Second, in this current study, the RHHI was positioned freehand based on anatomic landmarks, advised by the implant manufacturer, without a digital pre-operative planning. The authors agree with Alolabi et al. [8], intraoperative fluoroscopy may provide additional valuable information to confirm offset and varus/valgus of the implant. Finally, explanation could be patient selection, as some patients may have benefited more with a total shoulder arthroplasty.

Another limitation to this cohort study is the use of the “true” antero-posterior radiograph of the shoulder. Theoretically, the measurements could vary according the position of the arm or the scapula. Therefore, we only used the best 6-months or 1-year radiographs for postoperative measurements, which had better quality compared to direct postoperative radiographs. Moreover, Spiegl et al. [44] and Bouaicha et al. [45] showed that the CSA assessed on radiographs is equal to a computer tomography (CT) scan and superior to a MRI scan.

The modified LGHO was assessed in multiple studies on radiographs [10, 20, 21]; in literature, there is no study which compared the (modified) LGHO on radiographs compared to CT or MRI scan.

Many studies use the COR for hemi- and TSP arthroplasty on patient radiographs [8, 46–48]. Other studies used CT on cadaveric shoulders to assess the COR [40, 49, 50]. In literature, we found no superior evidence for CT or radiographs.

## Conclusion

In this study, we demonstrated that the CSA, LGHO, and COR are reliable radiologic measurement methods with high inter-observer agreement. The Global CAP resurfacing shoulder hemiarthroplasty will lead to overstuffing of the gleno-humeral joint in almost all shoulders. In contrast with the CSA and LGHO, we found a correlation between clinical failure and revision surgery in case of a deviation of the COR greater of 5 mm.

## Data Availability

The datasets during and/or analyzed during the current study available from the corresponding author on reasonable request.

## Abbreviations

AUC: Area under the curve
CAP: Conservative anatomic prosthesis
CFB: Change from baseline
CI: Confidence interval
COR: Center of rotation
CSA: Critical shoulder angle
CT: Computer tomogram
ICC: Intra-class coefficient
LGHO: Length of the gleno-humeral offset
MRI: Magnetic resonance imaging
OR: Odds ratio
RHHI: Resurfacing humeral head implant
ROC: Receiver operating characteristic
RSA: Reverse shoulder arthroplasty
SD: Standard deviations
SDD: Smallest detectable difference
SEM: Standard error of measurement
SPSS: Statistical package for the social sciences
TSA: Total shoulder arthroplasty
